# Physical and Mechanical Properties of Monomeric Alpha-Synuclein Provide Leads to Molecular Function

**DOI:** 10.1021/acs.jpcb.5c03200

**Published:** 2025-08-06

**Authors:** Katie Lynn Whitcomb, Kurt Warncke

**Affiliations:** Department of Physics, 1371 Emory University, Atlanta, Georgia 30322, United States

## Abstract

The activity of the intrinsically disordered protein, α-synuclein, in human brain neurons is associated with neurotransmitter storage, trafficking, and release, and its dysfunctional aggregation is linked to Parkinsons’s disease. To describe the as-yet unknown molecular function of α-synuclein, we address physical and mechanical properties of the isolated, monomeric human protein, by measuring the protein-coupled solvent dynamics detected by the electron paramagnetic resonance (EPR) spin probe, TEMPOL, colocalized in solvent regions around the protein, under temperature-controlled (200–265 K) ice-boundary confinement. The spin probe rotational correlation time at all temperatures is characterized by two components that are assigned to protein hydration water regions around nominally stable protein structure (slow motion; distal N-terminal and central domains) and to dynamically disordered regions (fast motion; C-terminal and proximal N-terminal domains). The equilibrium change from fast to slow motion components with decreasing temperature represents two sequential compaction processes of the protein. The processes are intervened by a dynamical disorder-to-order transition in the protein hydration solvent, which evidences the formation of stable, tertiary protein structure at a critical level of compaction. A model is presented, in which the dynamical macrostate reported by the spin probe at each temperature-dependent level of confinement is composed of an ensemble of structural microstates with common dynamical properties. The extrapolated room temperature free energy for compaction suggests facile modulation by *in vivo* confinement levels. The compaction and dynamical properties reveal molecular-mechanistic bases for function of α-synuclein *in vivo*.

## Graphical abstract

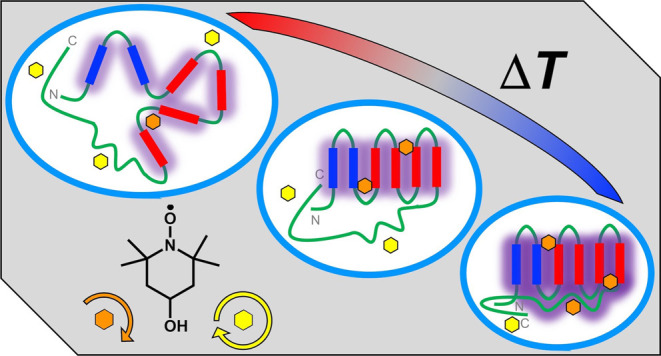

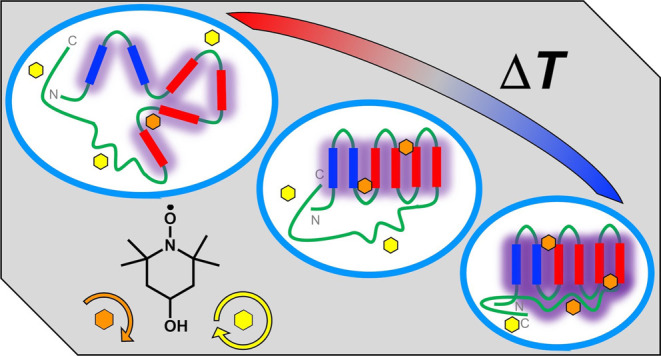

## Introduction

α-Synuclein is associated with the processes of intracellular neurotransmitter trafficking, release, and retrieval from the synaptic cleft in brain neurons.^1–3^ Aggregate oligomer and fibril forms of the 14.5 kDa protein are also a hallmark of Parkinson’s disease pathology in humans and animal models.^4,5^ Despite protracted effort, the molecular bases of α-synuclein function remain unspecified.^3^ Free, monomeric α-synuclein in solution is an intrinsically disordered protein (IDP),^6,7^ with a distribution of protein secondary and tertiary structures discernible by experiment,^8–11^ and detailed by computational techniques.^8,9,12–14^ Structural propensities in isolated, monomeric α-synuclein in solution are generalizable in terms of three canonical domains: N-terminal domain (NTD, residues 1–60; net positive charge, disordered proximal and structure-forming distal regions), central nonamyloid-β component (NAC, 60–95; β-strand and β-sheet formation) and C-terminal domain (CTD, 96–140; net negative charge and proline kinks in pseudorepeat sequence, dynamically disordered). Intramolecular interactions of the NTD and CTD in monomeric α-synuclein^8,9,11^ suppress intermolecular interactions of the hydrophobic NAC regions, that lead to protofibrils (β-sheet stacking, ∼5 Å diameter) and mature fibrils (cross-β-sheet interaction of two protofibrils, ∼10 Å diameter).^15,16^ The NTD-NAC can also nucleate oligomeric species.^17,18^ Projection of the dynamically disordered CTD from the core structure is a common feature of monomeric and all aggregate forms of α-synuclein, including the α-helical form (residues ∼8–80) adopted in the presence of phospholipid bilayer membranes.^19,20^

We have found that dynamic disorder in the protein-coupled solvent in oligomeric and fibrillar α-synuclein is remarkably robust, even resisting suppression in a vestigial component as the dynamically disordered regions are under increasing confinement.^21^ The controlled confinement is effected by the ice boundary that forms around the protein and grows with decreasing temperature (*T*) from 265 to 200 K in a frozen solution system.^22–25^ In this system, proteins and solutes, excluded from the advancing ice fronts during freezing, reside in interstitial fluid solvent regions, within the polycrystalline bulk ice. Inclusion of the electron paramagnetic resonance (EPR) spin probe, TEMPOL, also leads to its localization in the solvent region. TEMPOL reports on the dynamics of the solvent that interacts with, or is perturbed by, the protein (defined as protein-coupled solvent), through its rotational correlation time (τ_c_), and on the relative volumes of the phases, through the normalized proportions or weights (*W*) of the τ_c_-distinguished motional components. For cryotrapped folded, globular proteins, the ice boundary confinement is fine-tuned by using added cryosolvent (dimethyl sulfoxide, DMSO) to vary the thickness of a concentric shell of fluid aqueous-cryosolvent mesodomain phase (6–40 Å) between the ice boundary and the ∼6 Å thick protein hydration layer.^23,25^ In the absence of DMSO, the ice boundary severely restricts solvent motion in the hydration layer of folded, globular proteins.^26^ In dramatic contrast, the persistent low-temperature dynamics of the protein-coupled solvent phases around α-synuclein oligomers and fibrils^21^ arise from the properties of the intrinsically disordered regions of the α-synuclein protein, itself.^26^ The EPR spin probe thus reveals the degree of structure in the protein,^26^ and its influence on the surrounding solvent, which impacts binding interactions and function.^27^

To generate further leads to molecular foundations of α-synuclein function, we characterize the signature mechanical and physical properties of compaction and persistent solvent and coupled protein dynamics, previously described for oligomers and fibrils,^21^ in isolated, monomeric α-synuclein in the low-*T*, frozen solution system. Compaction with maintained structural disorder are general properties of IDPs,^28^ and low to moderate levels have been demonstrated for α-synuclein under different conditions of crowding *in vitro* ^29^ and in biological cells.^11,30,31^ In contrast to crowding, or the effect of excluded volume arising from a high total volume fraction of macromolecules,^32^ confinement, defined as the effect of excluded volume owing to an impenetrable boundary,^32^ such as the ice-wall cavity in our frozen solution system, offers resolution and quantification of the extent and thermodynamics of compaction, as well as inferences about the underlying microscopic protein and solvent structure, based on previous work.^8–14^ Confinement is also pertinent to the significant membrane boundary effects in the nerve terminal region, where α-synuclein functions.^1^ We find that monomeric α-synuclein displays a dynamical macrostate, or ensemble of microstate structures with common dynamics, at each temperature as compaction develops, with collapse to stable tertiary structure at a critical level of compaction, followed by collapse of the remaining dynamically disordered region with further decrease in temperature. The extrapolated room temperature free energy associated with compaction is accessible to *in vivo* modulation. These properties provide a molecular-mechanistic basis for functions of monomeric and presynaptic vesicle membrane-associated forms of α-synuclein in the nerve terminal region.

## Methods and Materials

### EPR Sample Preparation

All chemicals were obtained from commercial sources. Monomeric human α-synuclein was obtained from lyophilized powder (rPeptide, Watkinsville, Georgia, US; P/N S-1001-2). The lyophilized powder was initially suspended at 1 mg/mL in water (deionized, resistivity 18.2 MΩ cm), with brief vortex mixing (10–15 s). The suspension was passed through a filter (100 kDa cutoff; Amicon, Ultra-0.5 mL, Millipore) by using centrifugation, and the filtrate collected and used directly to prepare EPR samples. EPR samples included 0.5 mg/mL of α-synuclein in 10 mM potassium phosphate buffer (pH 7.4). TEMPOL was added from a stock solution to 0.02 mM. The total sample volume was 0.3 mL. Samples were transferred to EPR tubes (4 mm outer diameter; Wilmad-LabGlass, Buena, NJ, US), frozen by immersion in isopentane at 140 K, and stored in liquid nitrogen, prior to measurements.^25^

### Continuous-Wave EPR Spectroscopy

X-band CW-EPR spectroscopy was performed by using a Bruker E500 ElexSys EPR spectrometer and ER 4123SHQE X-band cavity resonator with temperature calibration and control, as described.^33^ The following acquisition parameters were used: microwave frequency, 9.5 GHz; microwave power, 0.2 mW; magnetic field modulation, 0.2 mT; modulation frequency, 100 kHz. Four spectra were averaged at each temperature.

### Transmission Electron Microscopy

EM grids (thick carbon, 400 mesh, copper grids; Electron Microscopy Sciences, Hatfield, PA, US) were cleansed by glow discharge. For each sample, a grid was placed on top of a 60 μL droplet for 5 min and then transferred to the top of a droplet of 2% uranyl acetate for 1 min. Grids were dried thoroughly before imaging. Images were obtained by using a Hitachi HT-7700 TEM, operating at 80 kV. TEM samples were prepared from the EPR sample after mixing of all components and prior to cryotrapping.

### EPR Line Shape and EPR Simulations

The X-band, continuous-wave EPR spectrum from the randomly oriented spin probe, TEMPOL, arises from the interactions of the unpaired electron spin (*S* = 1/2) with the external magnetic field (Zeeman interaction; defined by the **g** tensor) and the nitroxide ^14^N nuclear spin (*I* = 1) through the hyperfine interaction, which is defined by the ^14^N hyperfine tensor. The three spectral features correspond to electron spin–spin transitions (Δ*m*_s_ = ±1/2) among the three ^14^N nuclear spin states, *m*_I_ = 0, ±1.^34^ At higher temperatures, averaging of the dipolar hyperfine interaction by relatively rapid rotational motion of the spin probe resolves three spectral features, which are separated by the isotropic ^14^N hyperfine coupling constant. Slower rotation at lower temperatures leads to manifestation of the orientation-dependent dipolar hyperfine interaction, which broadens each *m*_I_ feature. The rotational correlation time, τ_c_, and component weights, *W*, which characterize the motional effects on the TEMPOL line shape,^35,36^ are determined by simulation of the EPR spectra. Methods for the simulation of nitroxide EPR spectra in the low-*T* frozen solution system, by using the program, EasySpin,^37^ and a common set of **g** and ^14^N hyperfine tensor principle values, have been described, in detail.^25^ The correlation times obtained from the simulations are in excellent agreement with those calculated for known solvent viscosity by using the Debye–Stokes–Einstein expression,^26^ and correspond to slow (τ_c_ → 10^–7^ s), intermediate, and rapid (τ_c_ → 10^–10^ s) TEMPOL tumbling regimes, that define the X-band motion-detection bandwidth.^35^

### Temperature Dependence of Spin Probe Rotational Correlation Time

The *T*-dependence of the rotational correlation time was addressed by using the molar form of the expression from Arrhenius rate theory:^38^

1 $$\tau_{c}=\tau_{c,0}10^{\left(E_{a}/2.3RT\right)}$$

where τ_*c*,0_ (units, seconds), *E*_*a*_ (kcal/mol), and *R* (1.987 cal/mol/K) are the Arrhenius rotational correlation time prefactor, activation energy, and gas constant, respectively. The expression implies that *E*_a_ and τ_c,0_ are *T*-independent over the measurement range. In this case, plots of log τ_c_ versus inverse *T* yield a linear relation with slope, $\frac{E_{a}}{2.3R}$ , and vertical axis intercept of τ_c,0_^–1^.

### Temperature Dependence of Spin Probe Mobility Components

The solvent phases in the low-*T* system are distinguished by different spin probe τ_c_ values. In this work, two τ_c_ values characterize the spin probe rotational mobility at each *T* value across the full range of *T* values. The two mobility components are labeled as slow (s; relatively slow motion, larger τ_c_ value) and fast (f; relatively fast motion, smaller τ_c_ value). The relative amplitudes of the spin probe rotational mobility components represent the relative volumes of the distinct solvent phases, in which the spin probe resides.^23,25^ The amplitudes, or weights, *W*, are normalized, so that the sum of the slow and fast weight components, *W*_s_ and *W*_f_, at each *T* value is unity. The spin probe EPR signal is conserved, as shown by repeated *T* increase and decrease cycles, performed with the same sample (Figure S2). Conservation of the spin probe EPR signal indicates that the slow solvent phase component (represented by *W*_s_) and the fast solvent phase component (represented by *W*_f_) undergo an equilibrium interconversion with changing *T* value,^21^ described by the simple equilibrium expression:

2 $$W_{f}⇌W_{s}$$

The corresponding Gibbs free energy difference for the direction of fast to slow solvent component conversion is

3 $$\Delta G=-2.3RT\;\log\left(\frac{W_{s}}{W_{f}}\right)$$

For the case of the standard van’t Hoff analysis, with *T*-independent enthalpy (Δ*H*) and entropy (Δ*S*), plots of $\log\left(\frac{W_{s}}{W_{f}}\right)$ versus inverse *T* yield a linear relation, with slope of $-\frac{\Delta H}{2.3R}$ , and vertical axis intercept of $\frac{\Delta S}{2.3R}$ .^38^

## Results and Discussion

### Ultrastructure of Monomeric α-Synuclein in EPR Samples

Transmission electron microscopy images of the isolated α-synuclein protein samples prior to cryotrapping show a population of spherical species with a fundamental unit diameter of approximately 5 nm (Figure 1A; Figure S1, control, grid background image). Particles of this shape and dimension have been assigned previously as monomers,^17,39^ and are consistent with the calibrated nominal pass-through particle size for the 100 kDa filter used to obtain the isolated α-synuclein sample.^40^ This dimension is also consistent with the ∼5 nm width of the α-synuclein monomer, as manifested in TEM images of single β-sheet protofilament (protofibril) structures, and the ∼10 nm width of fibrils, that are composed of two protofilaments interacting in the cross-β arrangement.^17,41^ Scrutiny of the TEM shows concatenated (linear, curved) and bunched particles of the fundamental dimension, that build, with decreasing presence, up to copy-numbers of ∼7. A minor population of larger aggregate particles of dimension ∼60 nm appear as collections of the larger oligomeric species. This observed distribution of monomeric and small-oligomeric α-synuclein species is expected for TEM of samples, as prepared here on the minutes time scale, from a moderately concentrated (35 μM) solution of monomeric α-synuclein, with gentle mixing and the flow conditions characteristic of filtering. Formation of larger oligomers and fibrils are processes that occur on the approximate time scale of days or longer, and at elevated temperatures (37 °C).^17,39^ Figure 1B shows that addition of DMSO to isolated α-synuclein does not significantly alter the fundamental size and distribution of the observed monomer, small-oligomer, and larger aggregate species.

**Figure 1:**
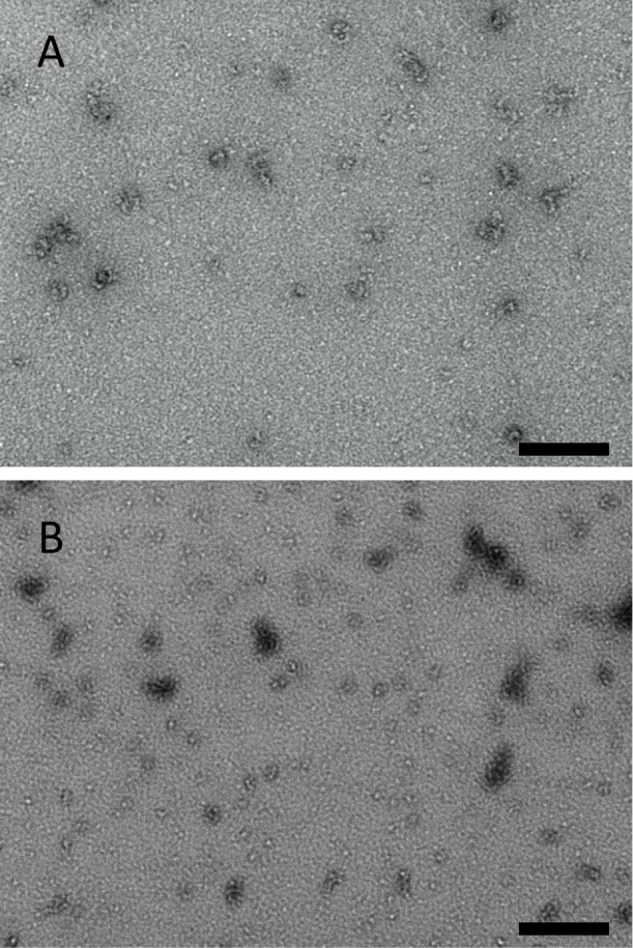
Transmission electron micrographs of isolated α-synuclein in the absence and presence of (A) isolated α-synuclein, −DMSO, and (B) isolated α-synuclein;, +DMSO. The condition shown corresponds to the EPR sample after mixing all components and prior to cryotrapping. Samples were prepared for electron microscopy, as described in Materials and Methods. Scale bar, 200 nm.

### Temperature Dependence of the TEMPOL EPR Spectrum in Samples of Monomeric α-Synuclein, in the Absence and Presence of DMSO

EPR spectra of TEMPOL in frozen solution samples of isolated α-synuclein in the absence and presence of DMSO, collected sequentially with increasing *T* from 220 to 265 K, progress from the rigid-limit, broad, powder-pattern line shape to the rapid tumbling, motionally narrowed line shape characteristic of the nitroxide spin probe (Figure 2).^25^ This general behavior, which is also observed for different classes of proteins in the low-*T* system,^26^ represents the increase in freedom of motion in the solvent associated with the protein, in which TEMPOL resides. For the isolated α-synuclein samples, the EPR line shapes of spectra collected during the sequential return in *T* from 265 to 200 K are identical to the line shapes collected during the sequential increase of *T*. Repetition of the ascending and descending *T* change protocol leads to a spectrum profile identical to Figure 2 (Figure S2). Therefore, *T* cycling does not change the structural and dynamical properties of the system. The independence of the spectrum-*T* correlation from the direction of *T* change for isolated α-synuclein contrasts with the dramatic dependence, or thermal hysteresis, observed for oligomer and fibril samples of α-synuclein.^21^

**Figure 2:**
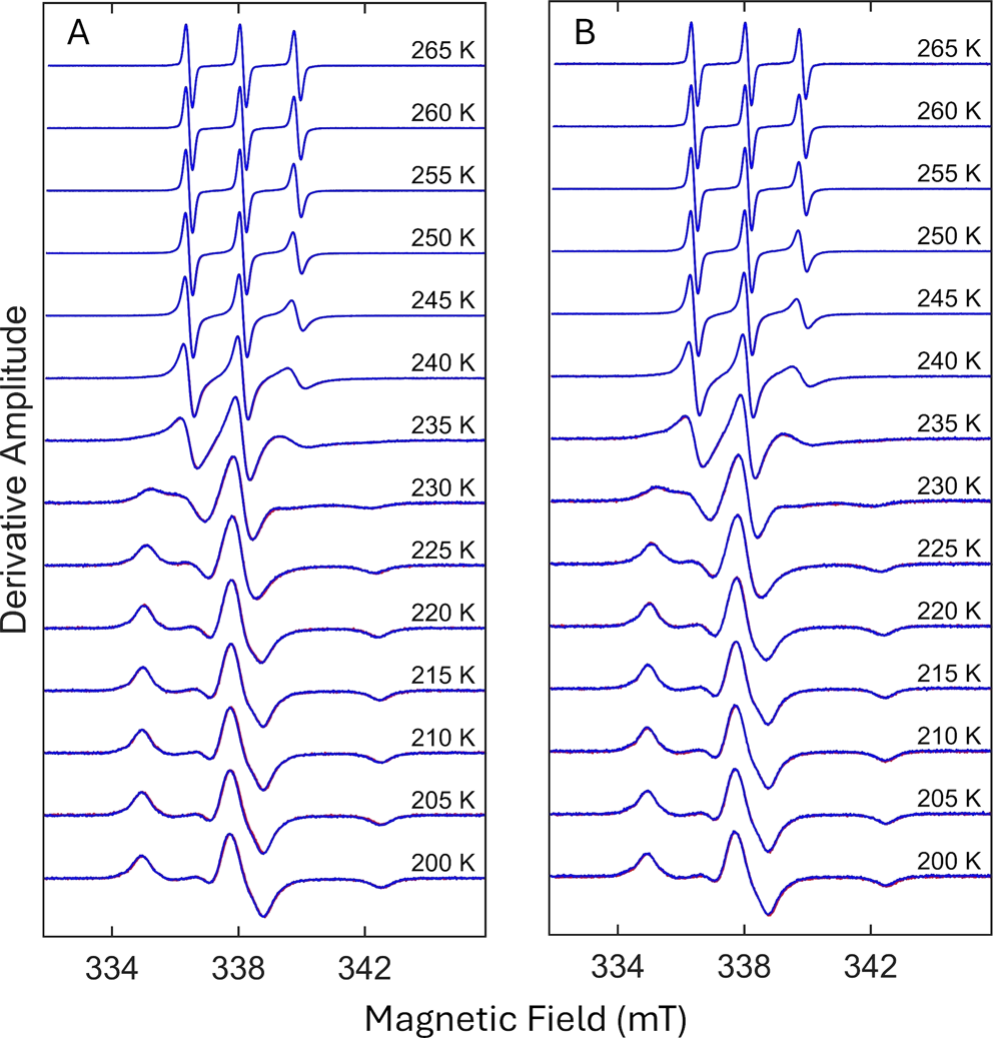
Temperature dependence of the TEMPOL EPR spectrum in samples of isolated, monomeric α-synuclein. (A) −DMSO. (B) +DMSO. Spectra represent increasing (red spectra) and decreasing (blue spectra) directions of sequential temperature change. Blue spectra overlay red spectra. Spectra are normalized to the central peak-to-trough amplitude.

### Temperature Dependence of the TEMPOL Rotational Correlation Times and Component Weights

The EPR line shapes of TEMPOL in samples of isolated α-synuclein are reproduced by EPR spectrum simulations at each *T* value by using two rotational mobility components. These two components are characterized by the rotational correlation time of the TEMPOL spin probe and corresponding normalized component weight: relatively slow mobility, log τ_c,s_ (logarithmic arguments are referenced to a value of 1 s), with corresponding normalized amplitude or weight, *W*_s_) and relatively fast mobility (log τ_c,f_, *W*_f_) (Figure S3 and S4, overlaid experimental and simulated spectra for spectra collected in directions of decreasing and increasing *T*; simulation parameters, Tables S1 and S2). Single component simulations do not match the line shapes, which are characteristic of TEMPOL spectra obtained in the low-*T* system in the presence of protein, as assessed comprehensively by comparison of single- and two-component simulations over the *T* range.^25^ The *T*-dependences of the log τ_c_ and *W* values for isolated α-synuclein in the absence and presence of DMSO are shown in Figure 3. Absence of thermal hysteresis in the log τ_c_ and *W* values is consistent with the observed identical EPR line shapes for spectra collected in the directions of increasing and decreasing *T* (Figure 2). The *W* values exhibit the trend of a dominant fast component in the high *T* range (≥255 K), that decreases with compensating slow component growth as *T* decreases. This trend of decreased *W*_f_ and rise of *W*_s_ was observed previously for oligomers and fibrils, and ascribed to compaction of the dynamically disordered regions with decreasing *T*.^21^ For the isolated α-synuclein samples, the decline in *W*_f_ with decreasing *T* is punctuated by an abrupt rise (−DMSO) or step (+DMSO), over approximately 240–235 K. Correspondingly, a kink positioned in the *T* range of the abrupt rise or step separates the two linear regions of the log τ_c,s_ dependence on *T*. These features suggest the presence of two dynamically distinct regimes of the protein-coupled solvent environment corresponding to the slow component: high-*T*, over 240–260 K, and low-*T*, over 220–235 K. Interestingly, the *T*-dependence of the fast component, log τ_c,f_, appears uniform over the *T* range.

**Figure 3:**
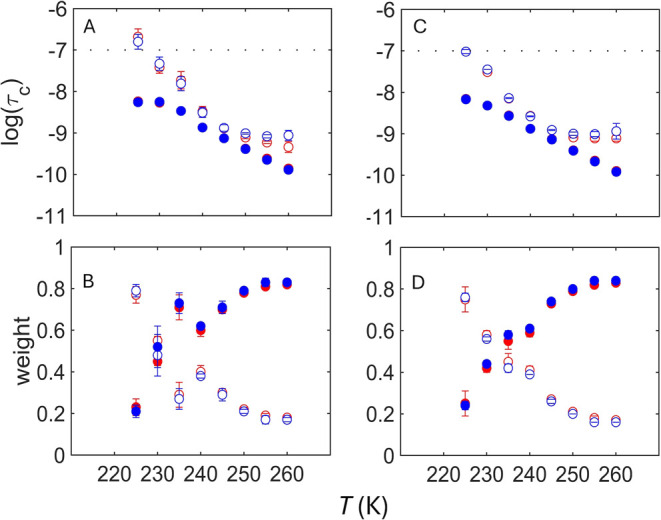
Temperature dependence of the rotational correlation time of TEMPOL and normalized mobility component weights for isolated α-synuclein. (A, B) Isolated α-synuclein, in the absence of DMSO. (C, D) Isolated α-synuclein, in the presence of DMSO. In each panel, open circles represent the slow component (log τ_c,s_, *W*_s_) and solid circles represent the fast component (log τ_c,f_, *W*_f_). The reference value for τ_c_ is 1 s. Spectra were acquired increasing (red) and decreasing directions (blue; overlaid). The horizontal line in panel A represents the upper limit on log τ_c_ for detection of tumbling motion. Error bars represent standard deviations for three separate determinations.

### Two Compaction Transformations and Intervening Dynamical Transition in Isolated α-Synuclein Are Revealed by Temperature-Controlled Confinement

Quantification of the spin probe rotational correlation time and component equilibrium processes is facilitated by examining their inverse temperature dependence (eqs 1 and 3). Plots of log τ_c,s_ versus inverse *T* for the −DMSO condition (Figure 4A; fitting parameters, Table S3) show two distinct linear relations. Comparison with Figure 4B shows that these correspond to two distinct linear regions of log­(*W*_s_/*W*_f_) dependence on inverse *T*. In the direction of decreasing *T*, these linear regions correspond to the compensating change from *W*_f_ to *W*_s_, representing the progressive conversion of dynamically disordered regions to immobile regions, as characterized previously in oligomeric and fibrillar α-synuclein.^21^ We therefore assign the two regions to a compaction process 1 (higher *T* range) and compaction process 2 (lower *T* range) (Figure 4B). In contrast to the two-stage behavior of log τ_c,s_, the properties of the dynamically disordered, protein-coupled solvent phase represented by log τ_c,f_ are uniform over the wide *T* interval (Figure 4A). This shows that the dynamically disordered domains in monomeric α-synuclein are maintained, even as their proportion is reduced by compaction.

**Figure 4:**
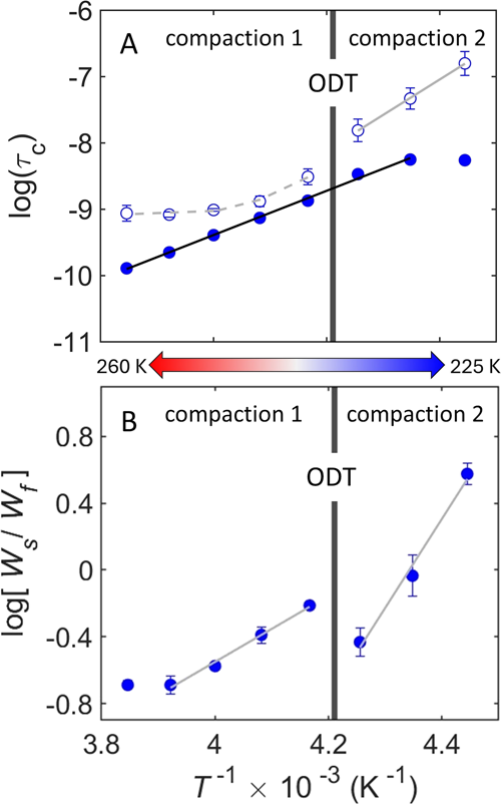
Inverse temperature dependence of the rotational correlation time of TEMPOL and ratio of normalized mobility component weights (as log_10_ value) for isolated α-synuclein in the absence of DMSO. (A) log τ_c,s_ (open symbol) and log τ_c,f_ (solid). The reference value for τ_c_ is 1 s. (B) log­(*W*_s_/*W*_f_). Results for the decreasing direction of sequential temperature change are shown. Temperature ranges of compaction processes 1 and 2 are indicated. The temperature of the order–disorder transition (ODT) is indicated by the vertical bar. Solid lines correspond to linear fits to the indicated regions (fitting parameters, Tables S3 and S4). The dashed line in panel A corresponds to the high temperature region of slow component *E*_a_ and log τ_c,0_ dependence on temperature. Error bars represent standard deviations for three separate determinations.

The presence of distinct slow components of spin probe mobility that correspond to compaction processes 1 and 2 requires that a transition exist between the compaction processes, that causes an abrupt change in properties of the phase represented by the slow mobility component. In fact, a dynamical, order–disorder transition in the hydration solvent layer around folded, globular proteins has been previously characterized in the low-*T*, frozen solution system, from the abrupt changes in *T*-dependence of slow-component spin probe motion^23,25,26^ and sample dielectric permittivity.^24^ This order–disorder transition (interval, <5 K) occurs at a temperature between 205 and 235 K for folded proteins, depending upon the degree of ice-boundary confinement.^22,23,25,26^ This transition has also been reported by others in different systems.^42–45^ The idiosyncratic rise in *W*_f_ at 235 K in the direction of decreasing *T* (Figure 3B), which causes the discontinuity in log­(*W*_s_/*W*_f_) at the juncture of compaction processes 1 and 2 (Figure 4B), is consistent with the characteristic exclusion of a proportion of spin probe from the hydration layer (slow component) of folded proteins into the surrounding mesodomain (fast component) upon hydration solvent ordering.^23^ We therefore assign the event, that occurs at a *T* value between compaction regimes 1 and 2, to an order–disorder transition (disorder-to-order, in the direction of decreasing *T*) in the hydration solvent phase associated with α-synuclein protein surface regions.

### Molecular Mechanism of Temperature-Dependent Confinement

Isolated, monomeric α-synuclein exists in a distribution of microstates of different structure.^8–11^ Computational approaches^8,9,12–14^ show species with different positions and levels of formation of isolated and mixed α- and β-secondary structure and attendant tertiary structure, which are primarily located in the NAC and distal NTD, along with disordered regions, primarily arising from the CTD and proximal NTD. Interactions of the NTD and CTD regions are prevalent.^8,9,11^ Thus, it is remarkable, given the distribution in α-synuclein microstate structures, that the spin probe-detected dynamics report just one uniform slow component from protein hydration solvent at each *T* value. This indicates that the hydration phases associated with the collection of secondary and tertiary protein structures in each microstate have closely similar dynamical character at each *T* value. For compaction process 1, we interpret the relatively shallow log τ_c,s_ dependence on *T* (Figure 4A) as caused by a *T*-dependence of the spin probe *E*_a_ and τ_0_ parameters, themselves [*E*_a_(*T*), τ_0_(*T*); depiction, Figure S5]. As the value of *T* decreases, *E*_a_(*T*) increases and log­[τ_0_(*T*)] compensatorily decreases (Figure S5). We propose that this trend of increasing *E*_a_(*T*) and decreasing log­[τ_0_(*T*)] is caused by the accumulation of tertiary structure regions as *T* decreases, which generates a growing, increasingly stable protein hydration region, represented by increasing *W*_s_ and compensating decrease of *W*_f_. As *T* further lowers, tertiary structure is consolidated, and the hydration matures, approaching a structure similar to the hydration layer supported by folded proteins.^23,26^ With the stable protein hydration structure structure formed, further decrease in *T* from 240 to 235 K elicits the characteristic disorder-to-order transition in the hydration layer.^23–26^ Below the transition, under compaction process 2, we propose that the NAC and distal NTD tertiary structure regions serve as sites for stabilizing the collapsing remaining dynamically disordered CTD and proximal NTD,^46^ with *T*-independent *E*_a_ and τ_0_. Our model for the behavior of the system over the full *T* range is depicted in Figure 5.

**Figure 5:**
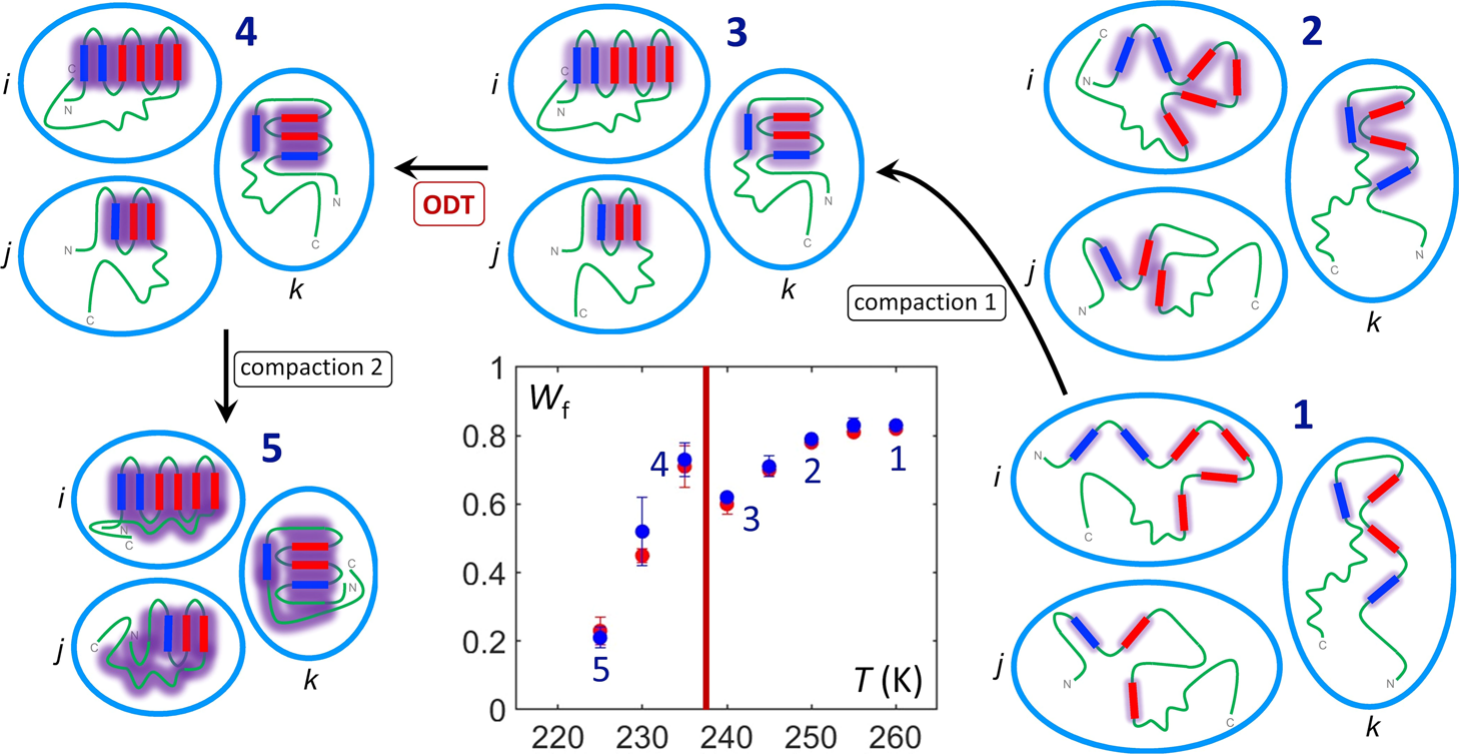
Depiction of the dynamical macrostate and structural microstate views of the sequential compaction 1, disorder-to-order transition and compaction 2 processes introduced by temperature-dependent confinement of monomeric α-synuclein. Macrostates **1**–**5** experience increasing compaction with lowering temperature, as quantified by the *W*_f_ (=1 – *W*_s_) values on the inset *W*_f_ versus *T* plot (from Figure 3). Three representative members of the microstate ensemble (***i***, ***j***, ***k***) depict development of protein structure from secondary to tertiary (blue, α-helix; red, β-strand; green, unstructured loop and dynamically disordered domains). The developing protein hydration layer is depicted as violet glow, with confinement-induced maturation in compaction process 1 (**1** → **3**) and stabilization of hydration regions (**3**) indicated by darkening and expanding glow. The ODT (**3** → **4**) is represented by further glow darkening of the maintained microstructures. Compaction process 2 entails collapse of residual dynamically disordered domains (**5**) onto the tertiary structure framework with maintained hydration layer properties. Over the full *T* range, the persistent dynamical disorder of the waning fast component is depicted by green microstate regions devoid of violet glow.

In summary, at each *T* value, an ensemble of *structural microstates* presents a common hydration solvent dynamical property, reported by τ_c,s_ of the hydration region-associated spin probe. Across the different α-synuclein structural microstates at each *T* value, the proportions of disordered, higher mobility protein regions also present a common dynamical property, reported by τ_c,f_. The properties reported by τ_c,s_ and τ_c,f_ represent a *dynamical macrostate*, with phase proportions represented by *W*_s_ and *W*_f_. The dynamical macrostate perspective, which is the direct EPR spin probe experimental observable, is consistent with the structural microstate perspective, which is supported by the distribution of α-synuclein protein structures characterized in independent experimental and theoretical studies.^8–14^

### α-Synuclein Protein-Coupled Solvent Dynamics in the Presence of DMSO

Overall, DMSO does not significantly influence the protein-coupled solvent dynamics of α-synuclein (Figure 3; inverse-*T* relations, Figure S6, fitting parameters Tables S3 and S4). This contrasts with the strong confinement-attenuating effect of DMSO on solvent dynamics around folded, globular proteins in the frozen solution system, associated with an aqueous-DMSO mesodomain shell, that surrounds the hydration layer and protein.^23,24^ Remarkably, DMSO addition does not significantly increase *W*_f_, or create a third solvent phase component, as expected if a separate aqueous-DMSO mesodomain was formed.^23,24^ Therefore, we propose that DMSO partially displaces water in both the protein hydration and dynamically disordered domains of α-synuclein. This model is consistent with replacement of the rise in *W*_f_ at 235 K (Figure 3B) with a short, approximately flat dependence in the presence of DMSO (Figure 3D). This indicates a reduction in the amount of characteristic spin probe exclusion from the protein hydration phase upon the transition from disordered to ordered,^23,24^ consistent with the presence of some DMSO in the hydration phase component of the IDP, which weakens water ordering. The absence of a strong confinement-relief effect of DMSO points to a dominant role of the α-synuclein protein, itself, in governing the dynamics of the disordered and hydration solvent phases.

### Thermodynamic Analysis of the Compaction Transitions

The two linear segments in the plots of $\log\left(\frac{W_{s}}{W_{f}}\right)$ versus inverse *T* (Figure 4B), that correspond to the compaction processes 1 and 2, were fitted by using eq 3 to obtain values of Δ*H* and Δ*S* (Table S4). In our model, these Δ*H* and Δ*S* values (and Δ*G* = Δ*H* – *T*Δ*S*) represent the conversion of dynamically disordered protein and coupled solvent into ordered domains, with decreasing *T* value. The fitted values of Δ*H* and Δ*S* include contributions from changes in self-interactions and freedom of the polypeptide, as well as from exclusion and relocation of water, that accompany compaction. The negative signs of both Δ*H* and Δ*S* are consistent with the proposed model of disordered domain compaction to form stable structural frameworks, and the accompanying aqueous solvent redistribution to form hydration and ice structure. Both of these processes involve a reduction in freedom (Δ*S* < 0) and formation of stable interactions (Δ*H* < 0). The increase in magnitudes of Δ*H* and Δ*S* in the low *T* compaction 2 regime represents an increase in both interactions and restriction of peptide and coupled solvent under higher confinement.

The extrapolated Δ*G* value corresponding to compaction in the range of standard (298 K) to physiological (310 K) temperature is estimated from the Δ*H* and Δ*S* parameters in the relevant compaction 1 regime (Table S4) as +3 kcal/mol. This value is only 5-fold larger than the available thermal energy (molar value, *RT* ≈ 0.6 kcal/mol), and modest relative to energies of noncovalent interactions *in vivo*. Volume exclusion by confinement contributes favorably to structural collapse by disfavoring the more extended protein microstates,^32^ overcoming the unfavorable Δ*G* values for compaction. For example, theoretical models for the free energy of folding of a protein of 100–200 residues in small cavities predict that confinement contributes 12–18 kcal/mol to folding as the cavity approaches the dimension of the folded protein.^32,47^ A cavity of dimension approximately 4-fold greater than the compact protein contributes 3 kcal/mol to the folding free energy.^47^ This suggests that a significant degree of compaction of α-synuclein in the neuron terminal region *in vivo* is accessible to modulation by confinement from the prominent synaptic vesicle and cell membrane boundaries.

### Molecular Bases for α-Synuclein Function *in Vivo*

Significant progress has been made in identifying the locations of α-synuclein in the presynaptic nerve terminal region and its interactions with membranes and other proteins, but molecular details of function remain unclear.^3^ The physical properties of α-synuclein presented here provide leads to molecular function. The cytosolic monomer form of α-synuclein must satisfy the contrasting demands of stabilization against intra- and interprotein β-sheet formation leading to dysfunctional oligomers and fibrils,^8–11^ while maintaining the ability to sense the high-curvature synaptic vesicle membrane, and transform to the NTD-NAC α-helical, membrane-bound form.^19,20^ Our results support and extend the proposed model of α-synuclein monomer stabilization through intramolecular interaction of the CTD with the NAC and NTD.^8–11^ In the nerve terminal region, the significant confinement condition enforced by the membrane surfaces of the high-density synaptic vesicle pool, as observed by EM,^48^ would act to compact secondary to tertiary structure and favor collapse of the CTD onto the core NAC-NTD structure, further veiling the β-strand-forming regions necessary for oligomer and fibril formation. However, the persistent dynamical disorder of the partially compacted α-synuclein CTD, as demonstrated here, would promote attraction to the synaptic vesicle liquid phase created by the vesicle-associated protein, synapsin,^49^ enhancing the probability of the observed interaction and twining of the α-synuclein CTD with the disordered NTD of the synaptic vesicle-anchored, integral membrane protein, VAMP2 (vesicle-associated membrane protein 2; synaptobrevin 2).^2,50–52^ Consistent with this, α-synuclein and VAMP2 are approximately stoichiometric in the synaptic vesicle pool.^53^ We suggest that the initial, localizing interaction leads to the reported specific interaction of the α-synuclein residue 96–110 region with VAMP2,^52^ and that this unveils NAC-NTD regions in the close vicinity of the synaptic vesicle membrane surface. This event initiates and promotes the specific pathway of the crucial transformation to the α-helical form of α-synuclein, further driven by interaction of positively charged side chains in the NTD region with negative charges on phospholipid headgroups at the membrane surface,^19,20^ rather than the pathway to amyloid forms. Increased synaptic vesicle density in synuclein knockout cells^48^ is consistent with CTD extension from the vesicle surface,^19,20^ leading to the suggestion that the dynamically disordered CTD contributes to distinction of the reserve, intermediate, and active zone synaptic vesicle pools. Recruitment of α-synuclein and establishment of its membrane-associated α-helical form at the synaptic vesicle reserve pool stage has the important downstream consequences for its promotion of SNARE (soluble *N*-ethylmaleimide-sensitive factor-attachment protein receptor) complex assembly,^54,55^ and contribution to efficient fusion pore dilation and neurotransmitter cargo release,^56^ following SNARE-mediated synaptic vesicle fusion with the presynaptic cell membrane.

## Conclusions

The EPR spin probe reports on the protein-coupled solvent dynamics associated with monomeric α-synuclein, as the protein is compacted by ice-boundary confinement introduced by decreasing *T* from 265 to 200 K. Compaction proceeds in two steps. The facile, higher-*T* compaction 1 process involves the formation of stable tertiary structure assigned to NAC and distal NTD regions, which, despite the distribution of structural microstates in monomeric α-synuclein,^8–14^ manifests common macroscopic dynamical properties of protein hydration and dynamically disordered regions at each *T* value. Formation of stable tertiary structure during compaction process 1 leads to a characteristic order–disorder transition^23,24^ in the hydration solvent, followed by a distinct compaction process 2 at lower *T* values. Compaction process 2 is assigned to CTD and proximal NTD collapse onto the folded regions. During these processes, dynamical disorder persists in the vestigial free CTD and proximal NTD. The modest compaction 1 free energy indicates ready modulation of α-synuclein compaction in response to confinement, *in vivo*, suggesting that confinement by membrane surfaces in the nerve terminal region^3,48^ promotes functional collapse of free α-synuclein, securing NTD-NAC regions from exposure and interprotein interactions that lead to aggregation and dysfunctional oligomer and fibril forms.^8,9^ The persistent dynamics in the monomeric α-synuclein CTD is consistent with attraction to the synaptic vesicle liquid phase,^49^ where specific CTD residue 96–110 interactions with synaptic vesicle-anchored VAMP2 ^52^ lead to unveiling of the α-synuclein core near the membrane surface,^2,50–52^ assuring high-fidelity transformation to the functional, membrane-associated, α-helical form (residues 8–80),^19^ with radially directed^19,20^ CTD.^54,55^ The revealed physical properties of the α-synuclein protein thus provide a basis for aspects of α-synuclein molecular function.

## Supplementary Material



## Notes

Mean log τ_c_ and *W* values at different *T* values for isolated α-synuclein in the absence and presence of DMSO, for data collection in the direction of decreasing and increasing *T* (Tables S1, S2); activation energy and correlation time prefactor parameters from linear fits to specified regions of the log τ_c_ dependence on inverse *T* (Table S3); thermodynamic parameters from linear fits to compaction 1 and compaction 2 regions of the log­(*W*_s_/*W*_f_) dependence on inverse *T* (Table S4); transmission electron microscopy (TEM) of isolated α-synuclein and background (Figure S1); temperature dependence of the TEMPOL EPR spectrum in the presence of isolated α-synuclein, showing repetition of the spectrum collection protocol by using the same sample, in separate experiments (Figure S2); temperature dependence of the TEMPOL EPR spectrum in the presence of isolated α-synuclein and overlaid two-component EPR simulations for collection of EPR spectra in the direction of decreasing (Figure S3) and increasing (Figure S4) sequential temperature change; depiction of the proposed origin of the weak, curved log τ_c,s_ dependence on *T* for the slow-component at *T* values above the order–disorder transition (Figure S5); inverse temperature dependence of the rotational correlation time of TEMPOL and ratio of normalized mobility component weights for isolated α-synuclein in the presence of DMSO (Figure S6) (PDF)
