# Supplementary material for: Physical and Mechanical Properties of Monomeric Alpha-Synuclein Provide Leads to Molecular Function
Source: J Phys Chem B. 2025 Aug 6;129(33):8351–9. doi: 10.1021/acs.jpcb.5c03200 (PMC12376096; doi:10.1021/acs.jpcb.5c03200)
Supplement: Supplementary file 1 [file jp5c03200_si_001.pdf]

Physical and Mechanical Properties of Monomeric Alpha-Synuclein Provide Leads to Molecular Function

Katie Lynn Whitcomb and Kurt Warncke*

Department of Physics, Emory University, Atlanta, Georgia 30322

Supporting Information

Table of Contents

SI Figures

Figure S1. Transmission electron microscopy (TEM) of isolated α-synuclein and background.

Figure S2. Temperature dependence of the TEMPOL EPR spectrum in the presence of isolated α-synuclein, showing repetition of the spectrum collection protocol by using the same sample, in separate experiments (separate dates).

Figure S3. Temperature dependence of the TEMPOL EPR spectrum in the presence of isolated α-synuclein and overlaid two-component EPR simulations (red, dashed line), for collection of EPR spectra in the direction of decreasing sequential temperature change.

Figure S4. Temperature dependence of the TEMPOL EPR spectrum in the presence of isolated α-synuclein and overlaid two-component EPR simulations (red, dashed line), for collection of spectra in the direction of increasing sequential temperature change.

Figure S5. Depiction of the proposed origin of the weak, curved log $\tau_{c,s}$ dependence on T for the slow-component at T values above the order-disorder transition.

Figure S6. Inverse temperature dependence of the rotational correlation time of TEMPOL and ratio of normalized mobility component weights for isolated α-synuclein in the presence of DMSO.

SI Tables

Table S1. Mean logτ_c and W values at different T values for isolated α-synuclein in the absence of DMSO, for data collection in the direction of increasing T, and for data collection in the direction of decreasing T.

Table S2. Mean logτ_c and W values at different T values for isolated α-synuclein in the presence of DMSO, for data collection in the direction of increasing T, and for data collection in the direction of decreasing T.

Table S3. Activation energy and correlation time prefactor parameters from linear fits to specified regions of the log τ_c dependence on inverse T.

Table S4. Thermodynamic parameters from linear fits to compaction 1 and compaction 2 regions of the log(W_s/W_f) dependence on inverse T.

SI Figures

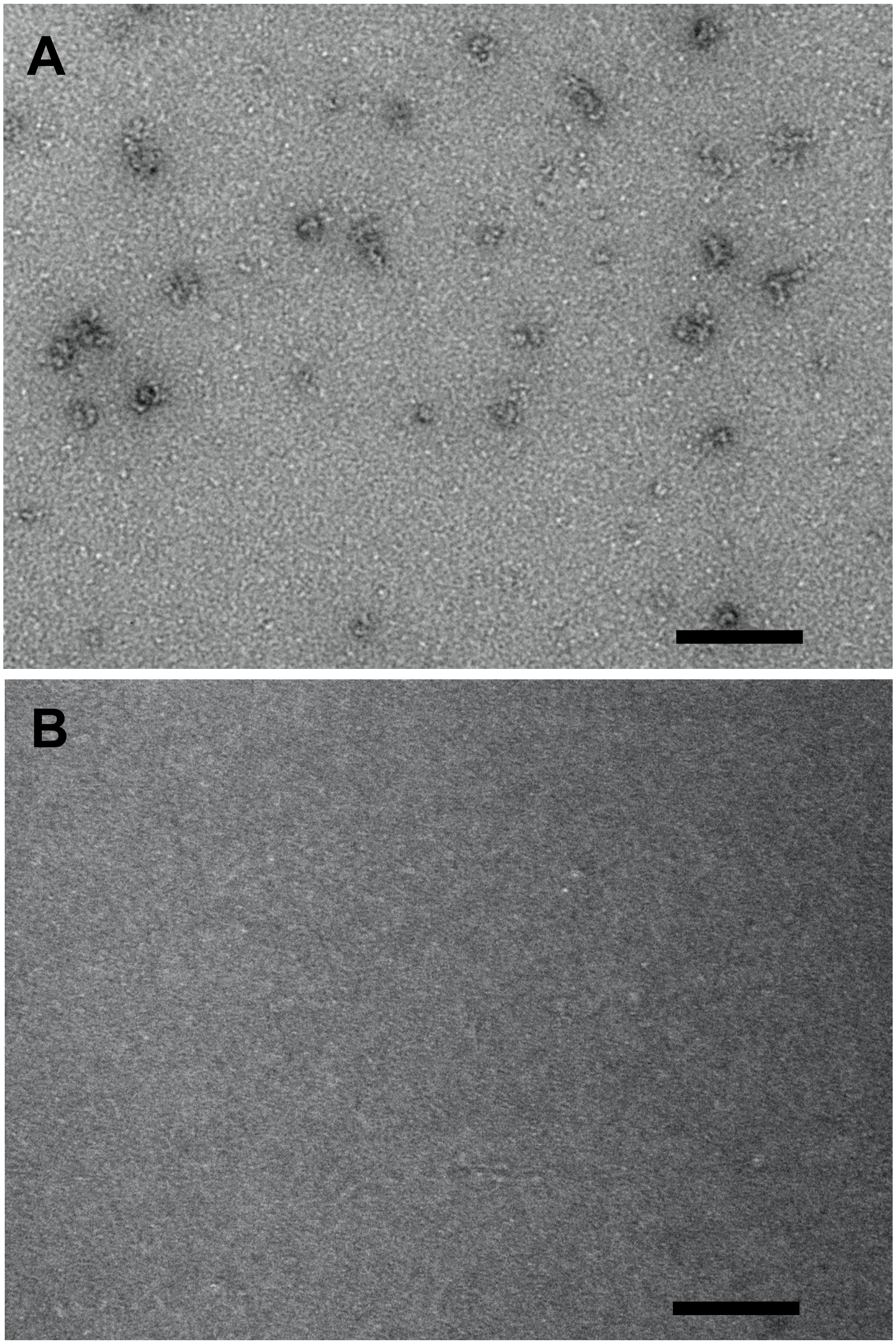

Figure S1. Transmission electron microscopy (TEM) of isolated α-synuclein and background. (A) Isolated α-synuclein, in the absence of DMSO (same image as presented in Figure 1). (B) Background of prepared EM grid only. Scale bar: 200 nm.

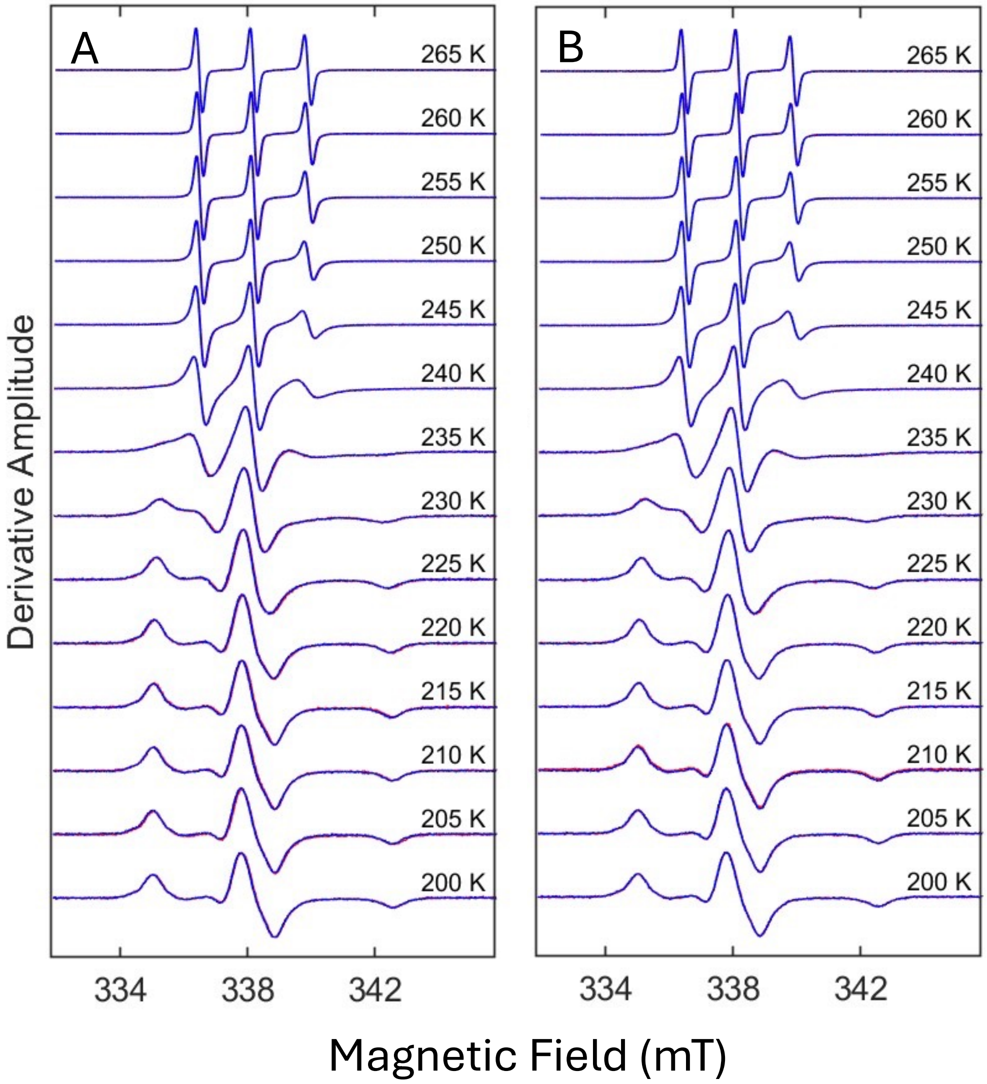

Figure S2. Temperature dependence of the TEMPOL EPR spectrum in the presence of isolated α-synuclein for collection of spectra in the directions of increasing (red spectrum) and decreasing (blue, overlaid spectrum) sequential temperature change, showing repetition of the spectrum collection protocol by using the same sample, in separate experiments (separate dates). (A) Original measurement. (B) Repeat measurement, performed on the same sample.

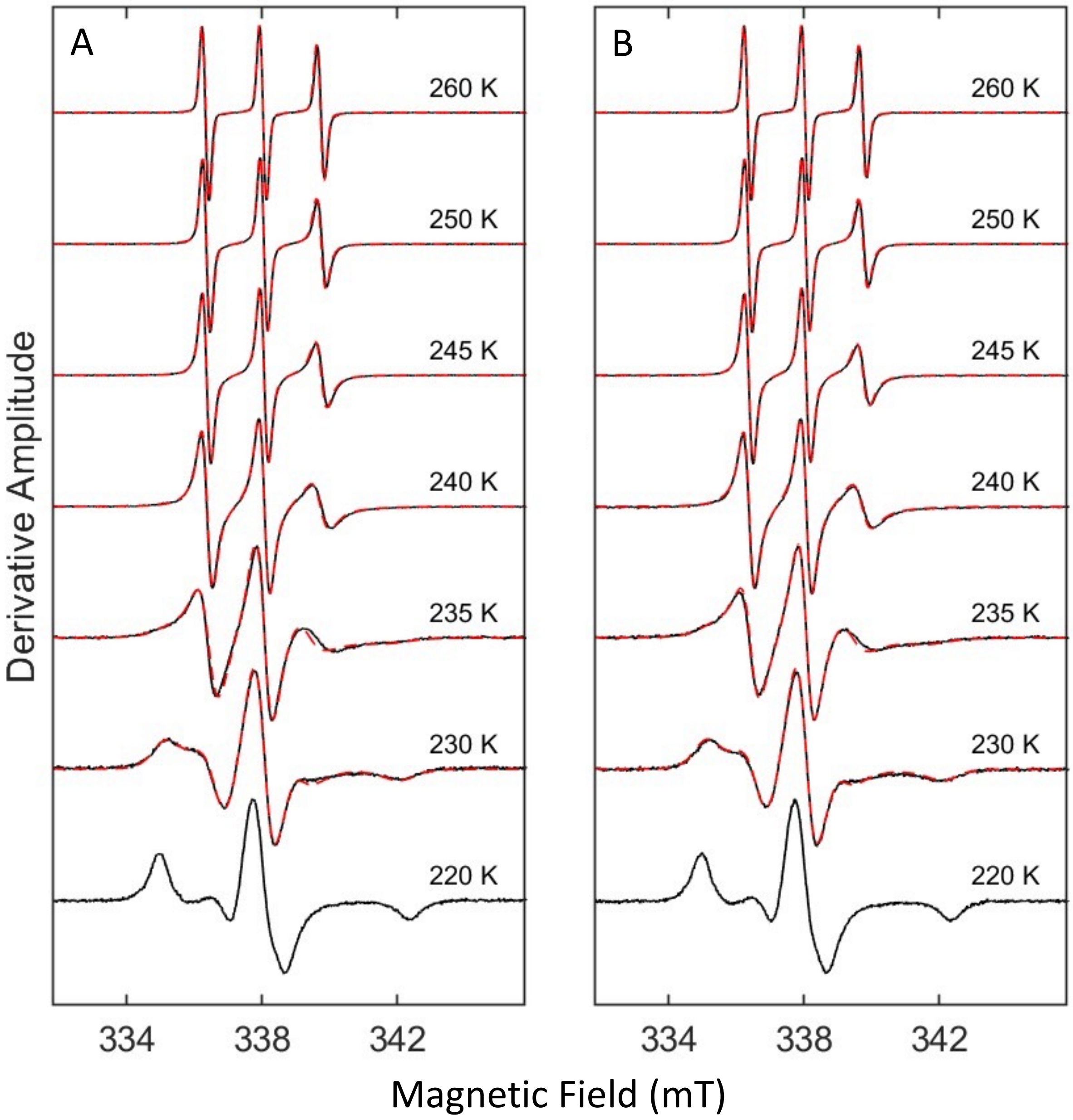

Figure S3. Temperature dependence of the TEMPOL EPR spectrum in the presence of isolated α-synuclein for collection of spectra in the direction of decreasing sequential temperature change, overlaid with two-component EPR simulations. (A) In the abence of DMSO. (B) In the presence of DMSO.

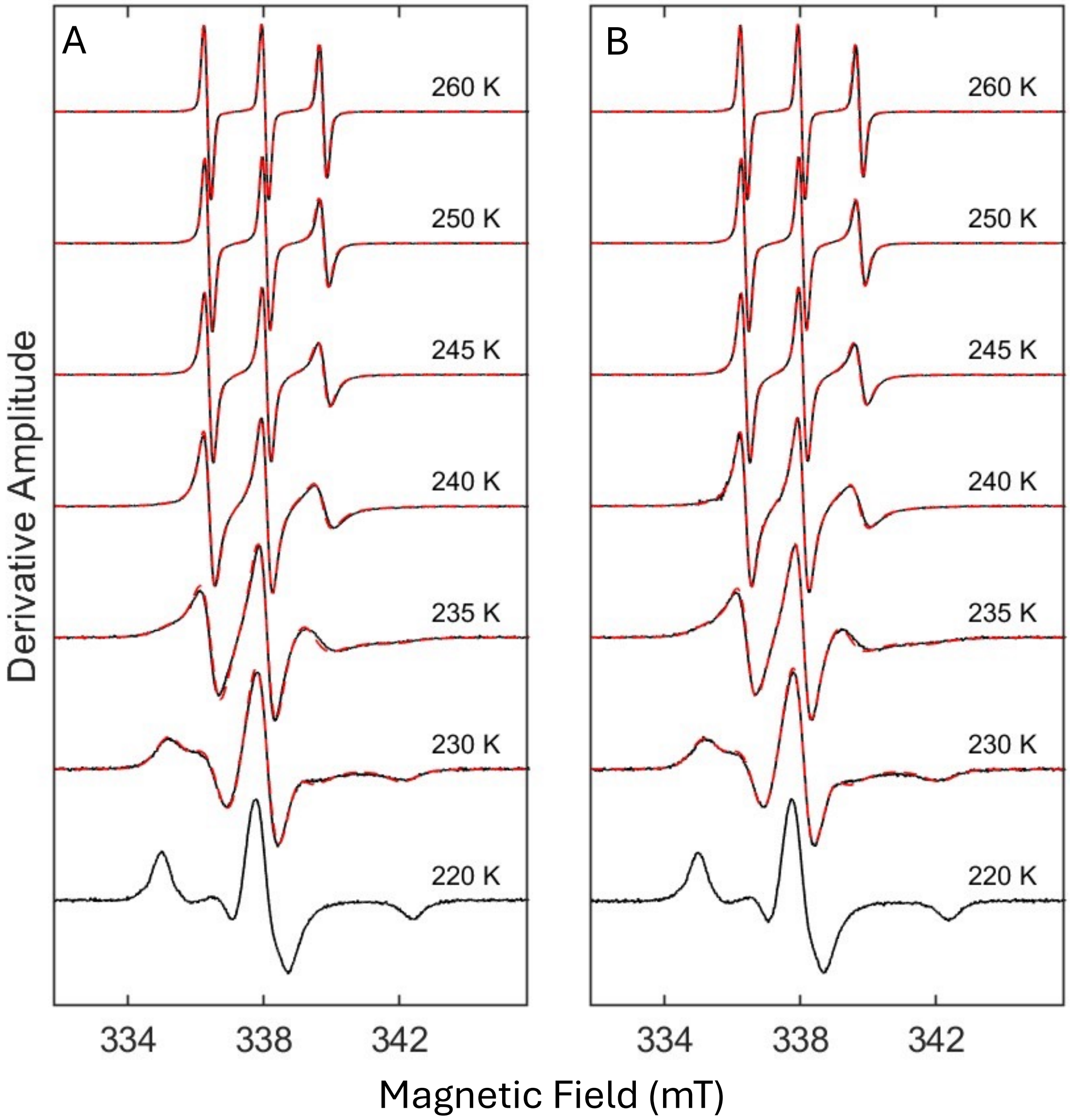

Figure S4. Temperature dependence of the TEMPOL EPR spectrum in the presence of isolated α-synuclein for collection of spectra in the direction of increasing sequential temperature change, overlaid with two-component EPR simulations. (A) In the abence of DMSO. (B) In the presence of DMSO.

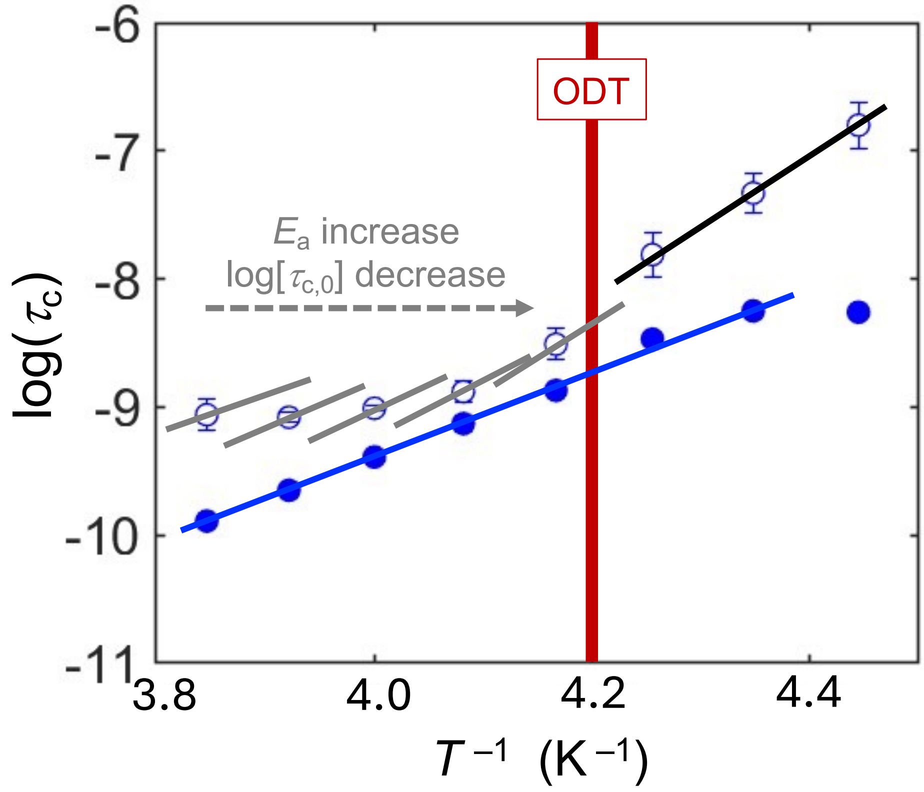

Figure S5. Depiction of the proposed origin of the weak, curved $\log\tau_{c,s}$ dependence on T for the slow-component at T values above the order-disorder transition (ODT; vertical red line). The origin is proposed to arise from a T-dependence of E_a and $\log\tau_{c,0}$ for the slow component in the high T, compaction 1 regime. The change in $E_a(T)$ and $\log\tau_{c,0}(T)$ is illustrated by representative short segments of Arrhenius dependence (Eq. 1), centered on each point, that would correspond to $E_a(T)$ [slope(T) = $E_a(T)/2.3R$] and $\log\tau_{c,0}(T)$ [y-intercept(T) = $\log\tau_{c,0}(T)$]. The segment slopes are chosen to be bounded by the slopes for the $\log\tau_{c,f}$ relation (highest T, lowest $1/T$ value) and $\log\tau_{c,s}$ relation (below ODT, highest three $1/T$ values). Reference value for τ_c is 1 s.

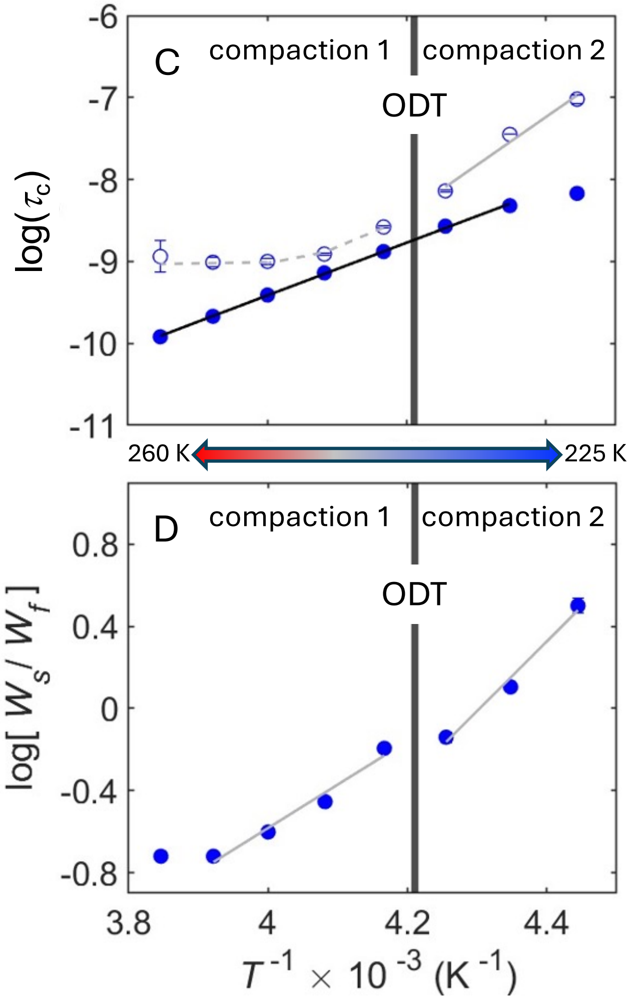

Figure S6. Inverse temperature dependence of the rotational correlation time of TEMPOL and ratio of normalized mobility component weights (as $\log_{10}$ value) for isolated α-synuclein in the presence of DMSO. (A) $\log \tau_{c,s}$ (open symbol) and $\log \tau_{c,f}$ (solid). Reference value for τ_c is 1 s. (B) $\log(W_s/W_f)$. Results for the decreasing direction of sequential temperature change are shown. Temperature ranges of compaction processes 1 and 2 are indicated. The temperature of the order-disorder transition is indicated by the vertical bar (ODT). Solid lines correspond to linear fits to the indicated regions (fitting parameters, Table S3, Table S4). Dashed line in panel A corresponds to high temperature region of slow component E_a and $\log \tau_{c,0}$ dependence on temperature. Error bars represent standard deviations for three separate determinations.

SI Tables

Table S1. Mean logτ_c and *W* values at different *T* values for isolated α-synuclein in the absence of DMSO, for data collection in the direction of increasing *T*, and for data collection in the direction of decreasing *T*. Values of $\tau_{c,s}$ and $\tau_{c,f}$ are referenced to the τ_c value of 1 s.

Increasing

T **(K)**	**log $\tau_{c,s}$**	**W_s**	**log $\tau_{c,f}$**	**W_f**
225	-6.68 ± 0.19	0.77 ± 0.04	-8.24 ± 0.06	0.23 ± 0.04
230	-7.42 ± 0.14	0.55 ± 0.02	-8.27 ± 0.05	0.45 ± 0.02
235	-7.73 ± 0.21	0.29 ± 0.06	-8.47 ± 0.07	0.71 ± 0.06
240	-8.48 ± 0.12	0.40 ± 0.03	-8.87 ± 0.05	0.60 ± 0.03
245	-8.89 ± 0.07	0.30 ± 0.02	-9.13 ± 0.04	0.70 ± 0.02
250	-9.11 ± 0.03	0.22 ± 0.01	-9.38 ± 0.04	0.78 ± 0.01
255	-9.23 ± 0.03	0.19 ± 0.01	-9.62 ± 0.04	0.81 ± 0.01
260	-9.34 ± 0.13	0.18 ± 0.01	-9.86 ± 0.04	0.82 ± 0.01

Decreasing

T **(K)**	**log $\tau_{c,s}$**	**W_s**	**log $\tau_{c,f}$**	**W_f**
225	-6.80 ± 0.18	0.79 ± 0.03	-8.26 ± 0.00	0.21 ± 0.03
230	-7.33 ± 0.16	0.48 ± 0.10	-8.25 ± 0.05	0.52 ± 0.10
235	-7.81 ± 0.17	0.27 ± 0.05	-8.47 ± 0.05	0.73 ± 0.05
240	-8.51 ± 0.12	0.38 ± 0.01	-8.87 ± 0.05	0.62 ± 0.01
245	-8.88 ± 0.08	0.29 ± 0.03	-9.13 ± 0.06	0.71 ± 0.03
250	-9.01 ± 0.02	0.21 ± 0.01	-9.39 ± 0.04	0.79 ± 0.01
255	-9.08 ± 0.04	0.17 ± 0.02	-9.65 ± 0.03	0.83 ± 0.02
260	-9.06 ± 0.12	0.17 ± 0.01	-9.89 ± 0.03	0.83 ± 0.01

Table S2. Mean logτ_c and W values at different T values for isolated α-synuclein in the presence of DMSO, for data collection in the direction of increasing T, and for data collection in the direction of decreasing T. Values of $\tau_{c,s}$ and $\tau_{c,f}$ are referenced to the τ_c value of 1 s.

Increasing

T (K)	log $\tau_{c,s}$	W_s	log $\tau_{c,f}$	W_f
225	-7.02 ± 0.05	0.75 ± 0.06	-8.16 ± 0.06	0.25 ± 0.06
230	-7.51 ± 0.02	0.58 ± 0.02	-8.32 ± 0.01	0.42 ± 0.02
235	-8.15 ± 0.07	0.45 ± 0.04	-8.56 ± 0.02	0.55 ± 0.04
240	-8.57 ± 0.01	0.41 ± 0.02	-8.88 ± 0.01	0.59 ± 0.02
245	-8.91 ± 0.00	0.27 ± 0.00	-9.13 ± 0.01	0.73 ± 0.00
250	-9.09 ± 0.01	0.21 ± 0.00	-9.40 ± 0.01	0.79 ± 0.00
255	-9.11 ± 0.07	0.18 ± 0.01	-9.65 ± 0.01	0.82 ± 0.01
260	-9.11 ± 0.07	0.17 ± 0.00	-9.90 ± 0.02	0.83 ± 0.00

Decreasing

T (K)	log $\tau_{c,s}$	W_s	log $\tau_{c,f}$	W_f
225	-7.02 ± 0.05	0.76 ± 0.02	-8.17 ± 0.03	0.24 ± 0.02
230	-7.45 ± 0.00	0.56 ± 0.01	-8.32 ± 0.01	0.44 ± 0.01
235	-8.14 ± 0.01	0.42 ± 0.02	-8.57 ± 0.01	0.58 ± 0.02
240	-8.58 ± 0.01	0.39 ± 0.00	-8.88 ± 0.01	0.61 ± 0.00
245	-8.91 ± 0.01	0.26 ± 0.01	-9.14 ± 0.01	0.74 ± 0.01
250	-9.00 ± 0.04	0.20 ± 0.00	-9.41 ± 0.01	0.80 ± 0.00
255	-9.01 ± 0.06	0.16 ± 0.00	-9.67 ± 0.01	0.84 ± 0.00
260	-8.94 ± 0.19	0.16 ± 0.00	-9.92 ± 0.04	0.84 ± 0.00

Table S3. Activation energy (E_a) and correlation time prefactor ($\log \tau_{c,0}$) parameters from linear fits to specified regions of the $\log \tau_c$ dependence on inverse T.[a] Values of $\tau_{c,s,0}$ and $\tau_{c,f,0}$ are referenced to the τ_c value of 1 s.

Condition	E_a (kcal/mol)	$\log[\tau_{c,s/f,0}]$
Slow Component[b]		
–DMSO	24 ±6.2	-31 ±5.9
+DMSO	27 ±1.5	-33 ±1.4
Fast Component[c]		
–DMSO	15 ±0.2	-23 ±0.2
+DMSO	15 ±0.2	-22 ±0.2

[a] Plots and fit lines are shown in Figure 4 (–DMSO) and Figure S6 (+DMSO). Fit corresponds to linear, first-order polynomial, performed by using MATLAB (Mathworks, Natick, MA). Fits were performed, individually, for each set of the triplicate data, for the direction of decreasing T, followed by calculation of average values of the parameters. Standard deviation corresponds to assumption of normal distribution for parameter values.

[b] Values correspond to the low-T, compaction 2 process, 225-235 K. The T-dependence of E_a and $\log(\tau_{c,s/f,0})$ in the high-T, compaction 1 region do not allow a fit to the Arrhenius relation.

[c] Values correspond to the T range, 230-260 K.

Table S4. Thermodynamic parameters from linear fits to compaction 1 and compaction 2 regions of the log(W_s/W_f) dependence on inverse T.[a]

	(kcal/mol)	(cal/mol/K)	(kcal/mol)
Condition	ΔH	ΔS	$T\Delta S$ [d]
–DMSO			
Compaction 1[b]	-9.4 ±0.91	-40 ±3.8	-9.8 ±0.93
Compaction 2[c]	-25 ±1.8	-107 ±8.2	-25 ±1.9
+DMSO			
Compaction 1[b]	-10 ±0.72	-44 ±2.9	-11 ±0.71
Compaction 2[c]	-16 ±1.8	-67 ±7.7	-15 ±1.8

[a] Plots and fit lines are shown in Figure 4 (–DMSO) and Figure S6 (+DMSO). Fit corresponds to linear, first-order polynomial, performed by using MATLAB (Mathworks, Natick, MA). Fits were performed, individually, for each set of the triplicate data, for the direction of decreasing T, followed by calculation of average values of the parameters. Standard deviation corresponds to assumption of normal distribution for parameter values.

[b] Values correspond to the high-T, compaction 1 process, 240-255 K.

[c] Values correspond to the low-T, compaction 2 process, 225-235 K.

[d] Representative values are calculated for T=245 K for compaction 1, and for T=230 K for compaction 2.
